# Recent Trends in Polymer Membranes: Fabrication Technique, Characterization, Functionalization, and Applications in Environmental Science (Part II)

**DOI:** 10.3390/polym18070895

**Published:** 2026-04-07

**Authors:** Gang Wei, Yan Wang

**Affiliations:** 1 School of Polymer Science and Engineering, Qingdao University of Science and Technology, Qingdao 266042, China; 2 School of Chemistry and Chemical Engineering, Qingdao University, Qingdao 266071, China

## 1. Introduction

Polymeric membranes have been widely applied in various fields such as adsorption, separation, energy conversion, chemical production, antibacterial, antifouling, and biomedicine due to their excellent porous and channel structure, as well as tailored separation performance, ease of operation, and low energy consumption. In terms of the polymeric membrane preparation technology, various methods such as phase inversion [1], interfacial polymerization [2], electrospinning [3], 3D printing [4], vacuum-induced filtration [5], template-assisted methods [6], and layer-by-layer assembly [7] have been widely applied. These techniques enable precise control over the membrane’s pore structure, thickness, surface roughness, and mechanical properties.

The functionalization of polymeric membranes is crucial to enhance their performance and extend their applications. Through molecular conjugation, chemical linking, physical grafting, plasma treatment, and nanomaterial compositing, the main properties and functions of polymeric membranes, including hydrophilicity, antibacterial activity, antifouling capability, catalytic activity, and selective transport properties have been improved to a great degree [8,9]. For instance, the combination of metal–organic frameworks [10], two-dimensional materials (graphene, MXene, and others) [11], self-assembled nanofibers [12], multifunctional nanoparticles [13], or antibacterial agents [14] into the polymeric membrane structure can not only enhance separation efficiency but also impart antifouling, antibacterial, or photocatalytic functions, enabling multifunctional integration of polymers and other components together.

The design of stimuli-responsive polymeric membranes also allows the membranes to dynamically adjust their pore size and flux in response to environmental stimuli, such as the tiny changes in temperature, pH, enzymatic level, light, electric fields, and others, providing new strategies for water treatment, drug release, and environmental monitoring [15]. In the field of environmental science, polymeric membranes have been extensively used for seawater desalination, wastewater purification, surface antifouling and antibacterial purposes, air cleaning, and others [16]. Nanofiltration, ultrafiltration, and reverse osmosis polymeric membranes have been widely applied to remove suspended particles, heavy metal ions, organic pollutants, and microorganisms from water, achieving efficient separation and water purification [17,18]. By selecting different polymer substrates and tailoring the pore structure and surface properties of membranes, polymeric membranes can be specifically designed to meet particular requirements in environmental science.

This collection is an extension of our previous Special Issue [19] entitled “*Recent Trends in Polymer Membranes: Fabrication Techniques, Characterization, Functionalization, and Applications in Environmental Science (Part I).*” In this editorial, we summarize the studies on the fabrication, functional regulation, and applications of polymeric membranes that were published in this special issue, and only a part of the results is excerpted from them.

## 2. Overview of the Published Articles

Twelve articles, comprising 1 review and 11 research articles, were included in the second part of this Special Issue.

In a review article (Contribution 1), Sahu et al. summarized recent advances in the fabrication of polymeric membranes for liquid separation, systematically discussing material innovations, fabrication strategies, and industrial applications. First, the mechanisms of membrane separation, including molecular sieving, adsorption, and electrostatic interactions, were introduced, and enable selective separation with high permeability. The authors then discussed commonly used polymers, such as cellulose, polysulfone (PSF), and polyethersulfone, for the fabrication of industrial polymeric membranes. In addition, several emerging membrane materials were highlighted, including mixed matrix membranes (MMMs) incorporating 2D materials or metal oxides, thin-film composite (TFC) membranes, and biopolymer-based membranes. These advanced membranes exhibit enhanced separation performance due to surface functionalization strategies such as surface grafting, nanomaterial hybridization, and layer-by-layer assembly. Functionalized polymeric membranes have been widely applied in industrial fields, including desalination, dye removal, heavy metal ion adsorption, ion separation and recovery, as well as applications in the chemical and food industries. Finally, the authors analyzed the challenges and limitations of polymeric membranes in industrial applications, emphasizing issues such as stability, fouling, degradation, and selectivity. This article provides a comprehensive overview of recent advances in polymeric membranes for liquid separation and is highly valuable for researchers in materials science, chemical engineering, environmental engineering, and nanotechnology.

Polymeric membranes are commonly prepared using methods such as phase inversion, template-assisted synthesis, interfacial polymerization, and controlled swelling of monolithic films. These techniques enable precise regulation of pore structure, membrane thickness, and surface properties, thereby meeting diverse separation requirements. By rationally designing the polymer composition and preparation process, the separation selectivity, permeation flux, and antifouling performance of polymeric membranes can be significantly improved. The study by Balasankar and co-workers (Contribution 2) introduced a facile fabrication method of superhydrophobic poly(carbonate) (PC) and poly(methyl methacrylate) (PMMA) membranes through anodized aluminum oxide (AAO) imprint molds as templates. The hierarchical structure of the AAO template was successfully transferred onto the polymer membrane surface using a mold imprinting technique, resulting in micropatterned polymer membranes with high surface roughness and excellent hydrophobicity. The study demonstrates that this template-assisted imprinting method is simple, cost-effective, structurally controllable, and scalable, highlighting its promising potential for applications in self-cleaning, antifouling, and anticorrosion surfaces. Through interfacial polymerization, Burts et al. (Contribution 3) prepared a novel TFC nanofiltration membrane with PSF, polyaniline (PANI), and polyamide (PA) as the support, interlayer, and selective layer, respectively. The results showed that the thickness, uniformity, and polymerization conditions of the PANI interlayer strongly influenced the pore structure, surface roughness, water flux, and salt rejection of the resulting TFC hybrid membranes. Importantly, the study demonstrated that membrane performance can be enhanced by tailoring the PANI interlayer, achieving both high permeability and improved selectivity. This interlayer-engineering approach combined with interfacial polymerization provides valuable guidelines for the design of functional polymeric membranes for water purification and industrial separation. In order to produce ultra-high-molecular-weight polyethylene (UHMWPE) ultrafiltration membrane, Basko and co-workers (Contribution 4) demonstrated a novel fabrication technique, called “controlled swelling of monolithic films”. Under hot pressing, UHMWPE powder was utilized to create a monolithic film, which was then proceeded under controlled swelling to form UHMWPE film. After extraction and drying, the UHMWPE film was dried to produce a porous ultrafiltration membrane. This study indicated that the molecular weight of the polymer affected the structure and properties of the formed UHMWPE membranes. For instance, it was hard to form membranes with the low-molecular-weight polymer, but a high molecular weight promoted the formation of dense, porous UHMWPE membranes with excellent mechanical properties, which could be further used for high-performance filtration and separation.

In addition, advanced fabrication techniques such as electrospinning and electrospraying can be employed to produce polymer membranes with controlled thickness and pore size. Electrospinning is typically used to create nanofibrous membranes with high porosity and flexibility, whereas electrospraying is well suited for forming particle-assembled membranes with tunable microstructures and facile surface functionalization. For instance, the study by Zhang et al. (Contribution 5) focused on the production of nanofibrous membranes via gas-assisted coaxial electrospinning (GACES). Compared to traditional coaxial electrospinning, the GACES could significantly adjust the flow field by introducing assisted gas, which could increase the fiber deposition area by 3 times, improve thickness by 2.3 times, and reduce the average fiber diameter by nearly 37%. This advanced electrospinning technique provides a theoretical and experimental basis for large-scale, uniform preparation of coaxial polymeric nanofiber membranes. In a recent study (Contribution 6), electrospraying has been utilized for the synthesis of jicama (*Pachyrhizus erosus*) starch particles. The effects of the hydrolysis degree of jicama starch on the formation and structure of electrosprayed particles were investigated. The results indicated that acidic hydrolysis reduced the gel viscosity and surface tension while increasing the conductivity, thereby altering the particle formation behavior during the electrospraying process. Although this study is not directly related to polymer membrane fabrication, the electrospraying technique and the insights gained from this control study provide valuable guidance for the design of biomass-based polymer membranes and their subsequent functional regulation, showing great potential for applications in environmental and food science.

The functional regulation of polymeric membranes is essential for optimizing their properties and performances in environmentally related applications. Surface modification, plasma treating, and incorporation of nanomaterials further enable polymeric membranes to exhibit enhanced adsorption, antifouling, catalytic, or stimuli-responsive behaviors.

Polymer modification is an important strategy for tailoring the structure and performance of polymeric membranes. Through chemical functionalization, the introduction of additional active groups can endow polymeric membranes with higher selectivity and flux, as well as improved mechanical stability. Papchenko and co-workers evaluated the CO_2_ capture performance of two commercial anion exchange membranes (AEMs), Fumasep^®^ FAA-3-50 (Fuel Cell Store, Bryan, TX, USA) and Sustainion^®^ X37-50 Grade RT (Dioxide Materials, Boca Raton, FL, USA) (Contribution 7). In these membranes, the polymer substrates were covalently functionalized with nitrogen-based cationic groups, where quaternary ammonium and imidazolium moieties were used to link with the polymer backbone, respectively. Through the CO_2_/CH_4_ adsorption tests, they demonstrated that both membranes exhibited good adsorption capacity towards CO_2_, and Sustainion^®^ had higher adsorption ability than Fumasep^®^. However, Fumasep^®^ showed higher CO_2_ diffusivity, revealing promising uses for moisture-swing air capture, biogas purification, and other innovative processes. In another case (Contribution 8), the effect of the polymer backbone’s side substituent on the performance of fabricated polymeric membranes was investigated. Comb-like polysiloxane was modified with side substituent (1-butanol, 1-propanol, and ethanol) and then fabricated to functional polysiloxane membranes. The obtained results indicated that the introduction of side groups could change the membrane’s hydrophobicity, solubility, selectivity, and mass transfer ability. Therefore, it is effective to enhance the separation efficiency of membranes in organic removal and water purification, by adjusting the side chain and molecular structure of polysiloxane.

Additionally, plasma treating of polymeric membrane surface can enhance their surface properties and biological activity. For instance, Ge et al. (Contribution 9) prepared a polyvinyl alcohol/polylactic acid (PVA/PLA) membrane by electrospinning, which was then treated with plasma to introduce more polar groups onto the membrane surface and further improve the hydrophilicity and surface energy. It was found that the plasma-treated PVA/PLA hybrid membrane exhibited increased liquid-enrichment capacity of 350% and shortened the coagulation time to 258 s. Meanwhile, the treated membrane presented 79% higher hemostatic ability than the untreated membrane.

Finally, the incorporation of functional nanoparticles, nanofibers, porous materials, and 2D materials can effectively modulate the pore structure, surface properties, and active sites of polymeric membranes, thereby expanding their potential applications across various fields. Cai and colleagues (Contribution 10) demonstrated that hybridizing Nafion membranes with sulfonated porous aromatic frameworks (PAFs) significantly improves their high-temperature proton conductivity. The sulfonated PAFs serve dual functions: they adsorb and retain water within the membrane and provide additional SO_3_H proton-conducting sites, which together stabilize proton transport channels under elevated temperatures. As a result, the PAF/Nafion composite membranes maintain superior proton conduction compared to pure Nafion at high temperatures. This study highlights the potential of sulfonated organic frameworks to enhance the performance of Nafion membranes for applications in energy conversion and environmental technologies. Guo and colleagues (Contribution 11) showed that functionalizing hydrophobic resin membranes with isothiazolinone (IS) and LDH–sodium pyrithione (SPT) composites effectively inhibits pipeline algal fouling. Chemical modification enhanced the hydrophobicity of the membranes, reducing initial algal attachment. Furthermore, IS and LDH-SPT acted as active anti-algal agents, suppressing algal growth and disrupting cell structures. Mechanistic analysis indicated a synergistic effect: the hydrophobic surface prevents adhesion while the chemical agents kill algae, together significantly lowering algae deposition and the risk of fouling in pipelines. In another study (Contribution 12), Liu et al. reported a strategy for preparing CuS nanoparticles through β-lactoglobulin (BLG) nanofiber templating and integrating them into MXene membranes to achieve photothermal antibacterial functionality. Their study showed that BLG nanofibers not only serve as templates to promote the uniform distribution of CuS nanoparticles but also enhance the structural stability of the MXene membrane. The composite membrane could rapidly heat under near-infrared light irradiation, killing bacteria through the photothermal effect while maintaining the membrane’s integrity and water transport performance. The study provides new ideas for the biomimetic synthesis of MXene-based functional membranes in antibacterial, water treatment, and biomedical fields.

## 3. Conclusions and Outlooks

In summary, the studies collected in this Special Issue demonstrate that the rational design of polymer composition, microstructure, and surface functionality plays a crucial role in advancing the performance of polymeric membranes. The integration of emerging materials, scalable fabrication techniques, and multifunctional modification strategies is expected to further expand the capabilities of polymeric membranes in water treatment, energy conversion, gas separation, environmental remediation, and biomedical applications.

With increasing global water scarcity, environmental pollution, and energy demand, the importance of efficient separation technologies has become increasingly prominent, providing unprecedented opportunities for the development of polymeric membranes. Here, we highlight several potential directions in this promising research field. First, the design of polymer molecules at the molecular level is suggested. The regulation of polymer chain and functional side groups are useful for tailoring the properties and microstructure of polymeric membranes. The membrane permeability, selectivity, bioactivity, and chemical stability can be precisely tuned through the introduction of ionic, hydrophilic, hydrophobic, biological, and rigid aromatic groups. Second, it is necessary to develop biomimetic polymeric membranes inspired by biological systems, which have the potential to achieve exceptional separation performance while maintaining high selectivity. Advanced fabrication techniques, such as 3D printing and biomimetic synthesis strategies, could be highly effective in achieving this goal. Third, smart polymeric membranes with stimuli-responsive properties can be designed and synthesized for environmental science and other applications. This goal can be achieved by incorporating responsive polymers and nanomaterials into the membrane systems, to create permeability and selectivity in response to external stimuli such as temperature, pH, light, enzyme, or electric fields. Fourth, multifunctional polymeric membranes with integrated functions such as separation, catalysis, antibacterial, and antifouling abilities are recommended. A potential solution for addressing this challenge is the use of multifunctional nanoparticles and nanozymes. Such multifunctional polymeric membrane systems can significantly enhance process efficiency and broaden their applications in environmental science, sensing, and biomedicine. Finally, artificial intelligence (AI) and data-driven approaches are increasingly being applied to the design, synthesis, and performance analysis of polymeric membranes, which can accelerate the discovery of novel membrane materials and the optimization of membrane structures. Additionally, machine learning methods are helpful for elucidating the relationships between membrane structure, function, and performance, thereby significantly accelerating the screening and development of advanced polymeric membranes.
